# Supplementary material for: Comparison of 0.12% Chlorhexidine and a New Bone Bioactive Liquid, BBL, in Mouthwash for Oral Wound Healing: A Randomized, Double Blind Clinical Human Trial
Source: J Pers Med. 2022 Oct 16;12(10):1725. doi: 10.3390/jpm12101725 (PMC9605239; doi:10.3390/jpm12101725)
Supplement: Supplementary file 1 [file jpm-12-01725-s001.zip › jpm-1914918-supplementary.pdf]

Supplementary Table S1: The sociodemographic characteristics of the patients involved in the study.

Sociodemographic characteristics	Number	Percentage
**Age, years**		
0–20	2	2.47%
21–30	4	4.94%
31–40	19	23.46%
41–50	32	39.51%
51–64	24	29.63%
**Sex**		
Male	37	45.68%
Female	44	54.32%
**Nationality**		
Spanish	54	66.67%
Other nationalities	27	33.33%
**Marital status**		
Married	57	70.37%
Single	21	25.93%
Divorced	3	3.70%
**Availability of social support**		
Yes	18	22.22%
No	63	77.78%
**Education level**		
Primary school	1	1.23%
High school	53	65.43%
University	20	24.69%
Postgraduate	7	8.64%
**Employment**		
Yes	67	82.72%
No	14	17.28%
**Socioeconomic status**		
Low	27	33.33%
Average	48	59.26%
High	6	7.41%

Supplementary Table S2: Healing Index as described by Landry et. al. 1988.

Healing index Score	Tissue color	Bleeding on palpation	Granulation tissue	Incision margin	Suppuration
1—very poor, Two or more signs are present	2: 50% of red gingiva	Yes	Yes	Not epithelized, with loss of epithelium beyond incision margin	Yes
2—poor	2: 50% of red gingiva	Yes	Yes	Not epithelized, with exposed connective tissue	No
3—good	25–50% of red gingiva	No	No	No exposed connective tissue	No
4—very good	< 25% of red gingiva	No	No	No exposed connective tissue	No
5—excellent	All pink tissues	No	No	No exposed connective tissue	No
